# Effect of *Enterobacter bugandensis* R-18 on Maize Growth Promotion Under Salt Stress

**DOI:** 10.3390/microorganisms13081796

**Published:** 2025-07-31

**Authors:** Xingguo Tian, Qianru Liu, Jingjing Song, Xiu Zhang, Guoping Yang, Min Li, Huan Qu, Ahejiang Tastanbek, Yarong Tan

**Affiliations:** Ningxia Key Laboratory for the Development and Application of Microbial Resources in Extreme Environments, College of Biological Science and Engineering, North Minzu University, Yinchuan 750021, China; 20227579@stu.nmu.edu.cn (X.T.); luckyq711@outlook.com (Q.L.); sjj2010745940@163.com (J.S.); yang_guoping@126.com (G.Y.); bkdlimin@126.com (M.L.); rosalie42@163.com (H.Q.); m18162018934@163.com (A.T.); m16672059258@163.com (Y.T.)

**Keywords:** abiotic stress mitigation, halotolerant microorganisms, maize, plant growth-promoting bacteria, salt tolerance

## Abstract

Soil salinization poses a significant constraint to agricultural productivity. However, certain plant growth-promoting bacteria (PGPB) can mitigate salinity stress and enhance crop performance. In this study, a bacterial isolate, R-18, isolated from saline-alkali soil in Ningxia, China, was identified as *Enterobacter bugandensis* based on 16S rRNA gene sequencing. The isolate was characterized for its morphological, biochemical, and plant growth-promoting traits and was evaluated for its potential to alleviate NaCl-induced stress in maize (*Zea mays* L.) under hydroponic conditions. Isolate R-18 exhibited halotolerance, surviving at NaCl concentrations ranging from 2.0% to 10.0%, and alkaliphilic adaptation, growing at pH 8.0–11.0. Biochemical assays confirmed it as a Gram-negative bacterium, displaying positive reactions in the Voges–Proskauer (V–P) tests, catalase activity, citrate utilization, fluorescent pigment production, starch hydrolysis, gelatin liquefaction, and ammonia production, while testing negative for the methyl red and cellulose hydrolysis. Notably, isolate R-18 demonstrated multiple plant growth-promoting attributes, including nitrogen fixation, phosphate and potassium solubilization, ACC deaminase activity, and indole-3-acetic acid (IAA) biosynthesis. Under 100 mM NaCl stress, inoculation with isolate R-18 significantly enhanced maize growth, increasing plant height, stem dry weight, root fresh weight, and root dry weight by 20.64%, 47.06%, 34.52%, and 31.25%, respectively. Furthermore, isolate R-18 improved ion homeostasis by elevating the K^+^/Na^+^ ratio in maize tissues. Physiological analyses revealed increased chlorophyll and proline content, alongside reduced malondialdehyde (MDA) levels, indicating mitigated oxidative damage. Antioxidant enzyme activity was modulated, with decreased superoxide dismutase (SOD) and peroxidase (POD) activities but increased catalase (CAT) activity. These findings demonstrated that *Enterobacter bugandensis* R-18 effectively alleviated NaCl-induced growth inhibition in maize by enhancing osmotic adjustment, reducing oxidative stress, and improving ion balance.

## 1. Introduction

Soil salinization poses a significant challenge to global sustainable agriculture, contributing to land degradation and economic losses [1]. In arid regions such as northwest China, saline soils are prevalent due to high evaporation rates [2]. Common salts include NaCl, Na_2_SO_4_, and others, with their composition and concentration varying regionally. Excessive salt accumulation disrupts plant ion homeostasis, leading to physiological drought and impaired nutrient uptake, ultimately stunting plant growth [3]. Therefore, improving saline soils, enhancing crop productivity, and increasing grain yields are critical challenges with major implications for sustainable agricultural development [4,5].

Currently, extensive research focuses on mitigating salt stress in plants to improve crop tolerance and productivity in saline-affected regions, thereby ensuring food security [6]. Maize (*Zea mays* L.), a staple crop, is moderately sensitive to salt stress, with optimal growth below 0.24% salinity and lethal effects at 0.485%. Given that salt stress inhibits maize growth, development, and physiological metabolism, developing effective strategies to enhance its salt tolerance is essential for agricultural sustainability [7].

To enhance crop adaptation to saline environments, salt-tolerant plant growth-promoting bacteria (ST-PGPB) may play a pivotal role in plant growth and stress resilience. ST-PGPB are root-associated bacterial communities that colonize the rhizosphere of plants in saline soils, where they enhance plant growth and ameliorate soil microenvironments [8]. Screening ST-PGPB, elucidating their mechanisms, and developing microbial inoculants have emerged as key research priorities for improving crop salt tolerance. Numerous studies have documented morphological and physiological improvements in plants following PGPB inoculation. Early research has demonstrated that specific microbial taxa, including *Bacillus pumilus*, enhance plant growth and salt tolerance through multifaceted strategies: improving soil microenvironments by solubilizing insoluble nutrients (e.g., phosphorus and potassium) and regulating soil pH as well as promoting nutrient uptake via the secretion of siderophores and auxins [9]. Recent advances have focused on ion homeostasis (e.g., K^+^/Na^+^ ratios), oxidative stress markers (e.g., lipid peroxides, MDA), and antioxidant enzyme activity to further unravel ST-PGPB-mediated salt tolerance [10,11].

For instance, *Enterobacter* strains isolated from cotton rhizospheres in Xinjiang’s saline soils exhibit salt tolerance and multifunctional PGP traits, such as N_2_-fixation, P-solubilization, and phytohormone secretion. Their demonstrated efficacy in cotton underscores their potential for salt-affected agroecosystems [12,13]. However, studies on PGPB-mediated salt tolerance in maize remain limited, and *Enterobacter* species have not been thoroughly explored in this context. In this study, we isolated *E. bugandensis* R-18 from saline-alkali soil in Ningxia, China. While Singh et al. [14] reported its extremotolerance, its agricultural applications remain underexplored. This study aims to investigate the ecological functions of this strain, particularly its role in enhancing salt tolerance and growth promotion in maize under NaCl stress. To evaluate whether strain R-18 exhibits salt-tolerant plant growth-promoting effects on maize, a pot experiment was conducted to address the following key questions: (1) Does strain R-18 improve maize growth parameters? (2) How does it regulate K^+^/Na^+^ distribution in seedlings? and (3) What are its effects on chlorophyll, proline, MDA, and antioxidant enzymes? The findings will provide novel insights into microbe-assisted stress resilience.

## 2. Materials and Methods

### 2.1. Experimental Samples

The soil sample for isolating and screening salt-tolerant growth-promoting bacteria was collected in the year 2023 from saline-alkali soil in Ningxia, China (106°29′ E, 39°02′ N; altitude 1055 m AMSL). The sampling site represents a typical saline-alkali agricultural field with the following characteristics: soil pH: 8.5; organic matter content (OM): 11.6 g/kg; K^+^: 141.37 mg/kg; Na^+^: 2457.76 mg/kg; Ca^2+^: 496.23 mg/kg; Mg^2+^: 102.54 mg/kg; SO_4_^2−^: 76.3 mg/kg; HCO_3_^−^: 313.33 mg/kg; Cl^−^: 666.67 mg/kg; CO_3_^2−^ was 0. The sampling depth was 0–30 cm. The collected soil sample was placed into a sterile bag and placed in a freezing box, which was then returned to the laboratory for low-temperature storage, intended for isolating and screening salt-tolerant growth-promoting bacteria. In this experiment, Ningdan 33 maize seeds, purchased from Ningxia Runfeng Seed Co., Ltd., China, were used for the pot experiment.

### 2.2. Medium and Reagent

The culture media and reagents used in this study, including Lysogeny broth (LB) medium, Ashby nitrogen-free medium, organic phosphorus medium, inorganic phosphorus medium, silicate bacteria medium, chromogenic agar for spores (CAS), ADF medium, and Salkowski reagent, were purchased from Haibo Biotechnology Co., Ltd. (Qingdao, China). Additionally, the plant chlorophyll content assay kit, proline content assay kit, and malondialdehyde (MDA) content assay kit were purchased from Cominbio Science & Technology Co., Ltd. (Suzhou, China). The superoxide dismutase (SOD) assay kit, peroxidase (POD) assay kit, and catalase (CAT) assay kit were purchased from Solarbio Science & Technology Co., Ltd. (Beijing, China).

### 2.3. Experimental Methods

#### 2.3.1. Isolation and Purification of the Bacterial Strains

Twenty grams of soil were aseptically weighed and homogenized with sterile distilled water at a 1:10 (*w*/*v*) ratio in a sterile conical flask. The suspension was vigorously vortexed and allowed to settle for 20 min at room temperature. The resulting supernatant was collected and subjected to serial dilution (10-fold increments) in sterile phosphate-buffered saline (PBS). Aliquots (100 μL) of the 10^−4^, 10^−5^, and 10^−6^ dilutions were spread-plated onto Lysogeny Broth (LB) agar plates and incubated at 37 ± 2 °C for 48 h under aerobic conditions. Distinct bacterial colonies, selected based on variations in morphology, pigmentation, and size, were purified via three successive streak-plating steps on fresh LB agar. Pure isolates were then inoculated onto LB agar slants and incubated for 48 h at 37 °C. Following incubation, the slants were labeled and stored at 4 °C for short-term preservation. To screen for halotolerant strains, purified isolates were cultured on LB agar supplemented with 5.0% (*w*/*v*) NaCl. Selected strains exhibiting salt tolerance were cryopreserved at −80 °C in LB broth containing 25% (*v*/*v*) glycerol for long-term storage.

#### 2.3.2. Physiochemical Characterization of Bacterial Strain R-18

The salt tolerance capacity of the strain was evaluated in LB liquid medium supplemented with graded NaCl concentrations (2.0%, 4.0%, 6.0%, 8.0%, and 10.0% *w*/*v*). The medium pH was adjusted to 7.0 ± 0.1 using 1 M NaOH or HCl prior to autoclaving (121 °C, 20 min). Bacterial inoculum (1 mL of standardized suspension) was aseptically transferred to 100 mL of sterile LB medium containing respective NaCl concentrations. Cultures were incubated at 37 ± 0.5 °C with continuous agitation (150 rpm) for 48 h in triplicate. Growth kinetics were monitored by measuring optical density at 600 nm (OD_600_) using a spectrophotometer. The maximum tolerance concentration was determined as the highest NaCl concentration supporting visible growth (OD_600_ > 0.1 compared to uninoculated control) [15]. To assess the alkali tolerance of the bacterial strain, LB liquid medium was adjusted to pH levels of 8.0, 9.0, 10.0, 11.0, and 12.0 using sterile 1 mol/L NaOH solution. Following sterilization at 121 °C for 20 min under moist heat conditions, bacterial inoculum (1 mL of standardized suspension) was aseptically transferred to 100 mL of sterile LB medium containing respective pH concentrations. The cultures were then incubated at 37 ± 2 °C with continuous shaking at 150 rpm for 48 h. Bacterial growth was subsequently evaluated by measuring the optical density at 600 nm (OD_600_) to determine the viable pH range for strain survival [16]. Morphological and physiological biochemical identification were conducted with reference to “Common Bacteria System Identification Manual” [17]. The target strains underwent identification tests including Gram staining and the methyl red test, fluorescence pigment test, V–P test, catalase activity, starch hydrolysis test, gelatin liquefaction test, ammonia production test, cellulose decomposition test, and citrate utilization test. Safety testing was performed using the blood agar plate method.

#### 2.3.3. Characterization of the Growth-Promoting Capacity of Strain R-18

Nitrogen fixation ability was determined using the ashby medium method, siderophore production ability was assessed using the CAS detection method, phosphorus solubilization ability was evaluated using the organic phosphorus medium and PKO inorganic phosphorus medium detection methods, potassium solubilization ability was determined using the silicate bacteria medium detection method, 1-aminocyclopropane-1-carboxylic acid (ACC) deaminase activity was measured according to the ADF medium detection method, and indole-3-acetic acid (IAA) production ability was determined using the Salkowski colorimetric method [9].

#### 2.3.4. Strain R-18 DNA Sequencing and Phylogenetic Analysis

After purification of the target strain, the genomic DNA extraction, PCR amplification with 16S rRNA-specific primers, purification of amplification products, and DNA sequencing were performed by Shengong Biotechnology Co., Ltd. (Shanghai, China). The PCR reaction system (25 μL) consisted of 10× PCR buffer, dNTP (each 10 mM), taq plus DNA polymerase (5 U/μL), 50 mM of MgSO_4_ (totaling 12.5 μL), primer 27F (AGAGTTTGATCMTGGCTCAG) and primer 1492R (AGAGTTTGATCMTGGCTCAG) (10 µmol/L), template (DNA) (1 μL each), and 9.5 μL of ddH_2_O to make up the total volume to 25 μL. The PCR reaction conditions were as follows: 95 °C for 5 min; 94 °C for 30 s, 57 °C for 30 s, 72 °C for 90 s, for 30 cycles; and 72 °C for 10 min. Molecular phylogenetic analysis was performed by retrieving homologous gene sequences (primarily 16S rRNA for bacterial taxonomic identification) from the NCBI GenBank database (https://www.ncbi.nlm.nih.gov/genbank/, accessed on 30 December 2024) using BLASTn (v2.13.0+), with stringent selection criteria requiring >97% sequence identity and high query coverage for reference sequences, followed by multiple sequence alignment using the ClustalW algorithm (v2.1) implemented in MEGA v11.0 software package (https://www.megasoftware.net/, accessed on 12 February 2025), which included subsequent manual refinement of gap regions to optimize alignment accuracy.

#### 2.3.5. Pot Experiment

(1) Preparation of the Bacterial Suspension

The selected strain R-18 was inoculated into LB liquid medium and cultivated on a shaker at 150 r/min until turbidity was observed. The culture was then transferred to centrifuge tubes and centrifuged at 10,000 r/min for 10 min to remove the supernatant. The resulting biomass was resuspended in sterile water to obtain a bacterial suspension with a concentration of 1 × 10^8^ CFU/mL.

(2) Different treatments of maize seedlings

Uniform-sized Ningdan 33 maize seeds were aseptically placed in a sterile beaker. The seeds were treated with 75% ethanol, stirred for 15–20 s, and then drained. Subsequently, an appropriate amount of 6% NaClO was added, and the seeds were intermittently stirred for 15 min for surface disinfection. Afterward, the seeds were washed with sterile water 3–5 times and soaked in sterile water at 4 °C for 10 h. The soaked maize seeds were then placed on 0.8% water agar medium and incubated in the dark at 25 ± 2 °C until the roots reached a length of 2–3 cm. An experiment was conducted to evaluate the growth-promoting effects of treatments on maize in hydroponic systems. The culture bottles were disinfected with 75% ethanol and filled with 450 g of sterile white stones. We initially investigated the growth performance of Ningdan 33 maize seedlings under varying NaCl concentrations through hydroponic pot experiments to determine the optimal salinity level for subsequent studies. In the experimental design, we established three treatment groups as follows: (1) a non-saline control group irrigated with Hoagland nutrient solution (control group; pH 6.73, EC 1916 μS/cm), (2) a saline stress group treated with Hoagland nutrient solution supplemented with NaCl (NaCl-treated group; pH 6.84, EC 12,420 μS/cm), and (3) a combined treatment group receiving Hoagland nutrient solution with 100 mM NaCl plus bacterial inoculation (NaCl+R-18-treated group). In the control and NaCl-treated groups, maize seeds were directly planted into the bottles after the emergence of the radicle, without prior immersion in bacterial suspension. In the NaCl+R-18-treated group, maize seeds with emerged radicles were immersed in bacterial suspension for 5 h before being planted into the bottles. Each bottle contained 4 seeds, with 3 biological replicates for each treatment. Each bottle was supplemented with 80 mL of Hogland nutrient solution, and the water level was marked. Sterile water was added to maintain the water level every 4 days. The maize seedlings were grown in a plant growth chamber under conditions simulating a mild summer climate, divided into five time periods as follows: 20 °C, 35% relative humidity, light intensity (9000 Lx) for 3 h; 25 °C, 35% relative humidity, light intensity (11,000 Lx) for 4 h; 28 °C, 35% relative humidity, light intensity (13,000 Lx) for 4 h; 25 °C, 35% relative humidity, light intensity (9000 Lx) for 5 h; 18 °C, 35% relative humidity, light intensity (0 Lx) for 8 h.

(3) Determination of various indexes of maize seedlings

After a two-week growth period of the maize seedlings, the optimal salinity level for the “Ningdan 33” maize seeds was evaluated. Seedlings subjected to different treatments were collected, and their growth parameters—including plant height, stem diameter, fresh and dry weights of stems and leaves, root length, as well as fresh and dry weights of roots—were measured to analyze salt tolerance effects [18]. The K^+^ and Na^+^ contents in the roots, stems, and leaves of the maize seedlings were determined using an inductively coupled plasma optical emission spectrometer (ICP-OES, Optima 2100DV, PerkinElmer, Waltham, MA, USA) [19]. The contents of chlorophyll, proline, and malondialdehyde (MDA) were determined using the corresponding assay kits (plant chlorophyll content assay kit, proline content assay kit, and malondialdehyde content assay kit) following the manufacturer’s instructions (Suzhou Cominbio Science & Technology Co., Ltd., Suzhou, China). The activity of superoxide dismutase (SOD) was determined using the nitro blue tetrazolium (NBT) method; the activity of peroxidase (POD) was measured using the guaiacol method; and the activity of catalase (CAT) was assessed using the ammonium molybdate colorimetric method [20,21,22].

### 2.4. Data Analysis

The raw data were statistically processed and preliminarily analyzed using Microsoft Excel 2010. A phylogenetic tree for strain R-18 was constructed with MEGA v11.0. Statistical analyses were performed using one-way analysis of variance (ANOVA) in IBM SPSS Statistics v26.0 (SPSS Inc., Chicago, IL, USA), with all graphical representations generated using GraphPad Prism software (version 8.4.3; GraphPad Software, San Diego, CA, USA). A *p*-value < 0.05 was considered statistically significant.

## 3. Results

### 3.1. The Salt and Alkaline Tolerance of Strain R-18

**Figure 1:**
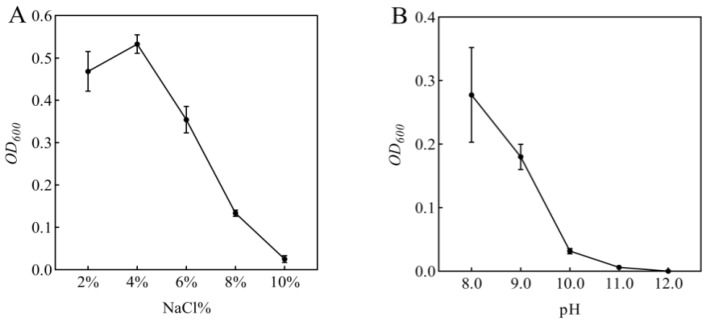
Growth response of strain R-18 under (**A**) NaCl stress and (**B**) pH gradients. The optical density (OD_600_) of strain R-18 was measured at 48 h, with data presented as mean ± SD (*n* = 3).

In this study, rhizosphere soil samples were collected and subjected to extensive bacterial screening through plating on LB agar medium. Based on morphological characteristics including colony shape, color, and size, 53 distinct bacterial isolates were successfully obtained and purified. Subsequent screening on LB medium supplemented with 5% NaCl yielded six bacterial strains exhibiting superior growth performance. These six strains were then evaluated for their plant growth-promoting effects under 100 mmol/L NaCl stress through pot experiments with maize seedlings. Notably, inoculation with isolate R-18 demonstrated significantly better improvement in maize growth parameters compared to other isolates (*p* < 0.05). Then, the bacterial suspension of strain R-18 (1 mL) was inoculated into 100 mL of LB liquid medium with varying NaCl concentrations or pH levels, followed by 48 h of shaking culture. The optical density at 600 nm (OD_600_) was measured, as shown in Figure 1A,B. The results showed that strain R-18 could grow normally in a NaCl concentration range of 2.0–10.0%, with the best effect at a NaCl concentration of 4.0%. After that, its activity gradually decreased. Strain R-18 could also grow in an environment with a pH of 8.0–11.0, and growth was significantly inhibited when the pH was greater than 10.0. It was preliminarily determined to be a salt-tolerant and alkali-tolerant bacteria, and subsequent experiments were conducted.

### 3.2. Assessment of Strain R-18 Morphology and Safety

**Figure 2:**
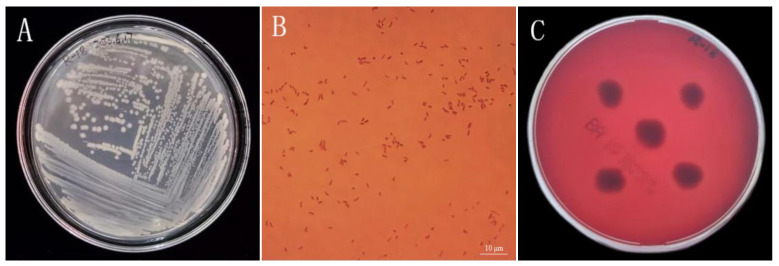
Morphological and phenotypic characterization of strain R-18. (**A**) Colony morphology of R-18 on an LB agar plate. (**B**) Gram-staining of R-18 (scale bar: 10 µm). (**C**) Hemolytic activity assay on blood agar.

After being cultured in LB solid medium for 48 h, the colony morphology of strain R-18 was observed. Its surface was moist, smooth, opaque, round, convex, milky white, and glossy and had neat edges. The colony diameter was approximately 1.0–1.2 mm (Figure 2A). Gram staining revealed a red, short, rod-shaped strain (Figure 2B). To assess the safety of strain R-18 by detecting its hemolysis, the blood agar plate method was used. No orange transparent zone appeared, indicating that the strain was normal (Figure 2C).

### 3.3. Performance Determination of Strain R-18

The results of the microbiological characteristics are presented in Table 1. The isolate was identified as a Gram-negative bacillus and tested negative for both the methyl red test and cellulose hydrolysis. However, it yielded positive results in the following biochemical tests: V–P test, catalase activity, citrate utilization, fluorescent pigment production, starch hydrolysis, gelatin liquefaction, and ammonia production. In the assessment of the plant growth-promoting abilities of strain R-18, it was found that this strain possesses nitrogen-fixing, phosphorus-solubilizing, and potassium-solubilizing capabilities. Additionally, it produced IAA and ACC deaminase, indicating its multifunctional nature as a plant growth-promoting bacterium.

### 3.4. Molecular Biological Characterization of Strain R-18

**Figure 3:**
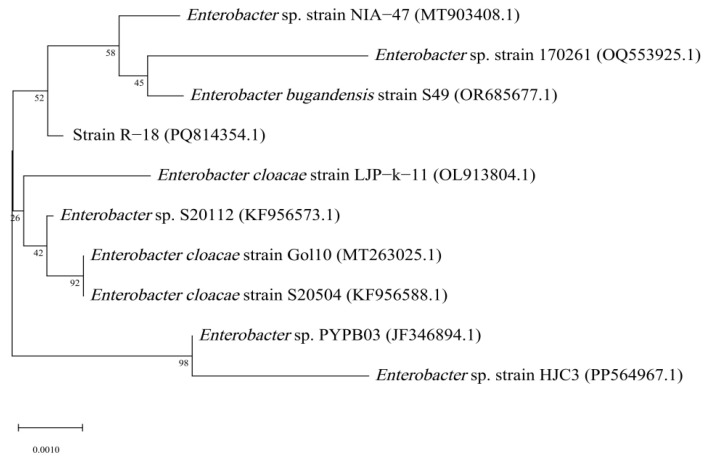
Phylogenetic tree of strain R-18 based on the 16S rRNA gene sequence. Numbers in parentheses are the GenBank accession numbers; Numbers at the branch nodes are bootstrap values, expressed as percentages of 1000 replicates; The scale bar indicates 0.2% nucleotide substitution.

The sequencing results of the PCR product of the 16S rRNA gene sequence revealed that the length of the 16S rRNA gene sequence of strain R-18 was 1476 bp. Through BLAST alignment analysis with nucleic acid sequence data in the GenBank database and using MEGA v11.0 to construct a phylogenetic tree of the strain, it was found that strain R-18 was closely related to *Enterobacter bugandensis*, with a similarity of 99.86%. Therefore, strain R-18 was preliminarily identified as *Enterobacter bugandensis* (Figure 3).

### 3.5. Effect of Strain R-18 on Maize Seedling Growth Index Under NaCl Stress

**Figure 4:**
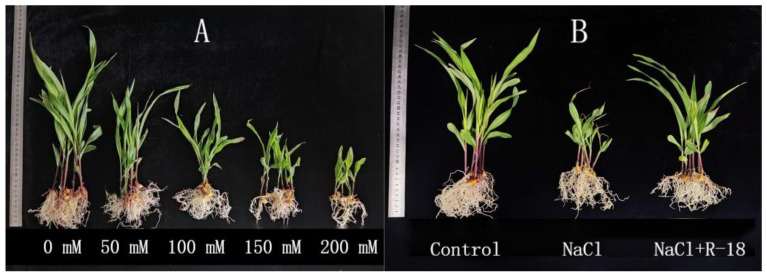
Growth of maize seedlings under NaCl concentrations of 0 mM, 50 mM, 100 mM, 150 mM, and 200 mM (**A**), and growth of maize seedlings under different treatment groups at 100 mM NaCl concentration (**B**).

The hydroponic screening revealed concentration-dependent responses of maize seedlings to NaCl stress (Figure 4A). No visible stress symptoms were observed in control (0 mM NaCl) and low-salinity (50 mM NaCl) treatments, with maize seedlings maintaining normal growth morphology. In contrast, severe growth inhibition and apparent tissue damage (leaf chlorosis and root browning) occurred at high salinity levels (150–200 mM NaCl). The 100 mM (0.58%) NaCl treatment elicited moderate yet statistically significant growth inhibition (*p* < 0.05), establishing this concentration as the optimal salinity level for subsequent experimental evaluations of strain R-18’s growth-promoting effects under salt stress conditions.

**Figure 5:**
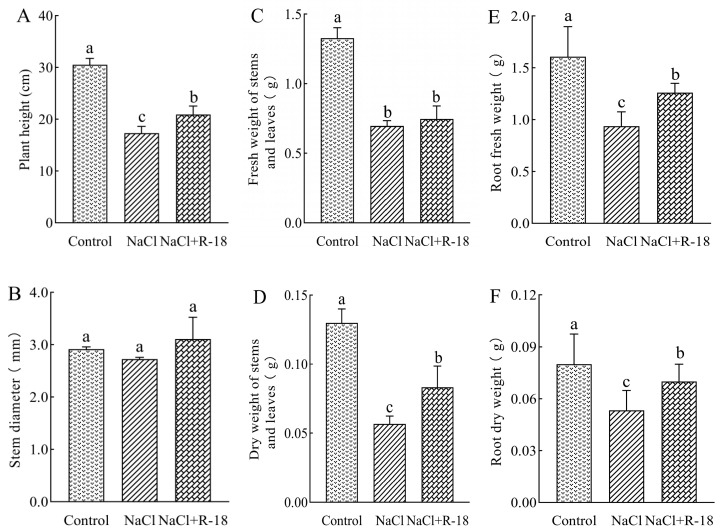
The effects of different treatments on growth indices of maize seedlings, where (**A**) was plant height, (**B**) was stem diameter, (**C**) was fresh weight of stem and leaves, (**D**) was dry weight of stem and leaves, (**E**) was fresh weight of root, and (**F**) root dry weight. Values = mean ± SD (*n* = 3). Different letters show significant differences (*p* < 0.05, ANOVA).

As can be seen from Figure 5, the growth indicators of maize seedlings in the control group were better than those in the NaCl-treated group. The plant height of the control group increased by 43.22% compared to the NaCl-treated group, indicating that 100 mM NaCl stress inhibited the growth of maize seedlings. As illustrated in Figure 5A,D–F, maize seedlings subjected to the NaCl+R-18 treatment exhibited significantly greater plant height, dry weight of stems, root fresh weight, and root dry weight compared to those in the NaCl-treated group, with increases of 20.64%, 47.06%, 34.52% and 31.25%, respectively (*p* < 0.05). Similarly, Figure 5B,C revealed that compared with the NaCl treatment group, the NaCl+R-18 treatment increased stem diameter and fresh weight by 14.04% and 11.47%, respectively. Furthermore, Figure 4B clearly reflected that inoculating with strain R-18 can effectively promote the growth of maize seedlings. It can be seen that inoculating with strain R-18 can significantly promote the growth of maize seedlings under salt stress.

### 3.6. Effect of Strain R-18 on the Salt Ion Level Under 100 mM NaCl Stress

**Figure 6:**
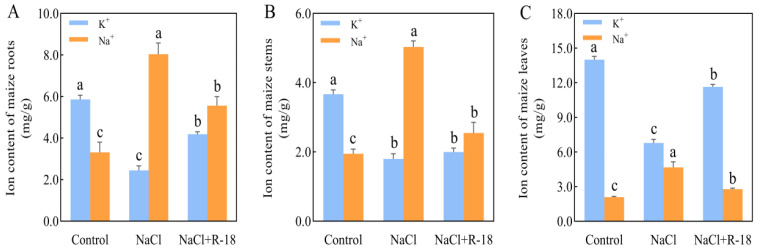
Distribution of potassium (K^+^) and sodium (Na^+^) ions in maize seedlings under differential treatments. (**A**) Root K^+^ and Na^+^ concentrations. (**B**) Stem K^+^ and Na^+^ concentrations. (**C**) Leaf K^+^ and Na^+^ concentrations. Values = mean ± SD (*n* = 3). Different letters show significant differences (*p* < 0.05, ANOVA).

The distribution and concentration of K^+^ and Na^+^ in roots, stems, and leaves of maize seedlings from different treatment groups were quantitatively analyzed after 14 days of growth, with the results presented in Figure 6. Significant differences were observed in the K^+^ and Na^+^ content in the roots of maize seedlings across various treatments. Root K^+^ content increased by 139.72% in the control and 70.98% in the NaCl+R-18-treated group relative to the NaCl-treated group. Conversely, the Na^+^ content was highest in the NaCl-treated group (Figure 6A). In terms of stem K^+^ content, the control group had the highest level, with the NaCl+R-18-treated group slightly higher than the NaCl-treated group. Notably, the Na^+^ content in the NaCl-treated group was significantly higher than that in the other two treatment groups, with the NaCl+R-18-treated group having a 49.34% lower Na^+^ content compared to the NaCl-treated group (Figure 6B). As is evident from Figure 6C, the K^+^ content in the leaves of maize seedlings in the control group and NaCl+R-18-treated group was significantly higher than that in the NaCl-treated group, being 2.1 times and 1.7 times higher, respectively. However, the Na^+^ content in the NaCl-treated group was 2.24 times and 1.68 times higher than that in the control group and NaCl+R-18-treated group, respectively (*p* < 0.05). Among the roots, stems, and leaves of maize seedlings, the K^+^/Na^+^ ratio was highest in the control group and lowest in the NaCl-treated group. Compared to the NaCl-treated group, the K^+^/Na^+^ ratio in the roots, stems, and leaves increased by 145.9%, 119.6%, and 188.2%, respectively, after inoculation with strain R-18. This indicates that, under the reference of the control group, the addition of NaCl caused the maize seedlings to absorb a large amount of Na^+^ in their roots, stems, and leaves, resulting in growth stress. However, after inoculation with strain R-18, all parts of the maize seedlings were able to effectively absorb K^+^, thereby inhibiting excessive absorption of Na^+^ and hindering maize growth. Among them, the absorption and distribution of K^+^ and Na^+^ in the leaves were most prominent. Therefore, subsequent experiments will mainly focus on using maize leaves to detect the response of strain R-18 to various indicators of maize seedlings under NaCl stress, in order to determine whether it has a salt-tolerant and growth-promoting effect on maize.

### 3.7. Effect of Strain R-18 on Chlorophyll, Malondialdehyde, and Proline Content in Maize Seedlings Under 100 mM NaCl Stress

**Figure 7:**
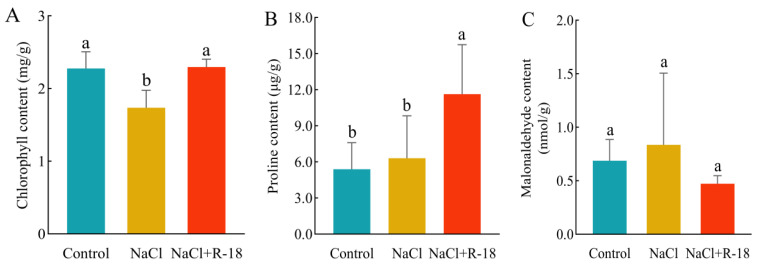
Physiological indicators of maize seedlings under different treatments. (**A**) Chlorophyll content. (**B**) Proline concentration. (**C**) Malondialdehyde (MDA) level. Values = mean ± SD (*n* = 3). Different letters show significant differences (*p* < 0.05, ANOVA).

In Figure 7A, the chlorophyll content represented the sum of chlorophyll-a and chlorophyll-b. Compared to the control group, the chlorophyll content in the NaCl-treated group of maize seedlings slightly decreased. After inoculation with strain R-18, the chlorophyll content increased by 32.24%. In Figure 7B, under salt stress, the proline content in the leaves of maize seedlings in the NaCl-treated group accumulated significantly, while the proline content in the control group was relatively low. The proline content in the leaves of maize seedlings in the NaCl+R-18-treated group increased by 84.13%, thereby enabling them to cope with the damage caused by NaCl stress. In Figure 7C, the MDA content in the leaves of maize seedlings in the NaCl-treated group was significantly higher than that in the control group; the MDA content in the NaCl+R-18-treated group decreased. This indicated that inoculating with strain R-18 can mitigate the impact of NaCl stress on the chlorophyll level of maize seedlings. The elevated proline content conferred protective effects on maize seedlings under NaCl stress, while the decreased MDA content attenuated lipid peroxidation in cellular membranes. This reduction in membrane permeability prevented the influx of deleterious compounds, thereby minimizing cellular damage.

### 3.8. Effect of Strain R-18 on Antioxidant Enzyme Activity Under 100 mM NaCl Stress in Maize Seedlings

**Figure 8:**
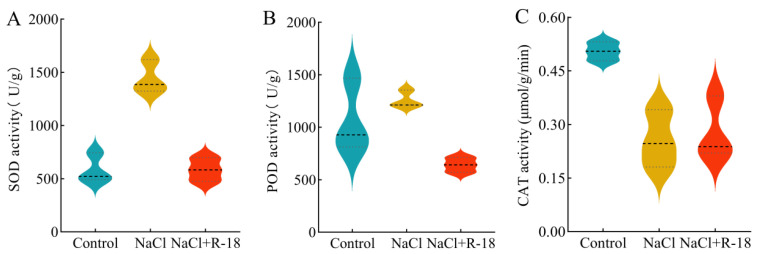
The antioxidant enzyme activity of maize seedlings under different treatments, where (**A**) was superoxide dismutase activity, (**B**) was peroxidase activity, and (**C**) was catalase activity. The probability density of data distribution (width), median (thick dashed line), and IQR (fine dotted line) are marked.

Figure 8 presents the results of the antioxidant enzyme activities in maize seedling leaves under 100 mM NaCl stress with different treatments. As is evident from Figure 8A,B, due to the 100 mM of NaCl stress, the SOD and POD activities in maize seedling leaves increased compared to the control group. However, after inoculation with strain R-18, the SOD and POD activities in maize seedling leaves significantly decreased compared to the NaCl-treated group, by 59.44% and 49.06%, respectively. Nevertheless, under 100 mM of NaCl stress, the CAT activity in maize seedling leaves significantly decreased compared to the control group. After inoculation with strain R-18, the CAT activity in maize seedling leaves increased by 11.6%, as shown in Figure 8C (*p* < 0.05). This indicated that inoculating with strain R-18 can alleviate oxidative damage in plants and mitigate the growth inhibition phenomenon caused by NaCl stress in maize seedlings.

## 4. Discussion

PGPBs are increasingly recognized as a nature-based solution for sustainable agriculture, particularly under abiotic stress conditions. Salinity, a major abiotic stressor, exerts detrimental effects on cellular organisms [23]. Under high-salinity conditions, elevated osmotic pressure drives the efflux of unbound water molecules from cells, resulting in cytoplasmic hyper-concentration. This process disrupts cellular functions and may ultimately lead to cell death. While all organisms face these challenges, PGPBs employ unique adaptive strategies that concurrently benefit host plants: bacterial osmolyte synthesis stabilizes both microbial and plant cellular structures under dehydration; exopolysaccharide secretion forms protective biofilms that reduce Na^+^ influx to plant roots; and ACC deaminase activity downregulates stress ethylene signaling in plants. These tripartite mechanisms explain why PGPB inoculation outperforms innate plant adaptation alone [24]. In this context, PGPBs have emerged as a promising strategy for both remediating saline soils and enhancing plant salt tolerance [25]. This study isolated *E. bugandensis* R-18 from saline-alkali soil, demonstrating significant halotolerant and alkaliphilic capabilities with growth observed at NaCl concentrations of 2.0–10.0% and pH ranges of 8.0–11.0. Recent studies have confirmed its presence in terrestrial ecosystems, particularly in saline-alkali, arid, and low-gravity environments. Notably, Arora and Jha [26] isolated *E. bugandensis* WRS7 from the rhizosphere and investigated its drought tolerance mechanisms that alleviate the detrimental effects of water stress in wheat. Numerous studies have demonstrated that PGPBs can enhance plant growth through various mechanisms, including biological nitrogen fixation, solubilization of insoluble potassium and phosphorus into plant-available forms, and production of IAA and ACC deaminase [27,28,29]. The isolate WRS7 exhibited multiple plant growth-promoting traits, such as nitrogen fixation and phosphate solubilization. Additionally, it produced siderophores, synthesized phytohormones (e.g., IAA and gibberellic acid), secreted exopolysaccharides, and showed ACC deaminase activity. Similarly, the isolate R-18 possessed comparable plant growth-promoting characteristics; these functional similarities with WRS7 suggest that strain R-18 may influence crop growth by modulating endogenous phytohormones or providing exogenous hormonal compounds.

*E. bugandensis* R-18 exhibited multifunctional plant growth-promoting (PGP) traits, including phosphate solubilization and the production of IAA and ACC deaminase. These characteristics classify it as a PGPB and indicate its potential as a microbial biostimulant. According to Rouphael and Colla [30], microbial biostimulants are defined as microorganisms that enhance nutrient use efficiency, stress tolerance, or crop quality, independent of their nutrient content. Notably, PGPB strains that directly benefit plants—through improved nutrient acquisition (e.g., N, P, Fe) or phytohormone regulation—are increasingly recognized within this category [31,32]. The ability of strain R-18 to alleviate salt stress and enhance maize growth aligns with the biostimulant functions of improved stress tolerance and nutrient use efficiency. Current studies have demonstrated that the pathogenicity of *Enterobacter* species exhibited significant strain specificity rather than being a universal trait at the species level [33]. Environmental isolates—particularly those derived from plant rhizosphere—typically lack clinically associated virulence factors, as their survival strategies are more oriented toward plant interactions and environmental stress adaptation rather than the infection of animals or humans [34,35]. Additionally, recent studies have revealed that only a small fraction of rhizospheric *E. bugandensis* strains carry single antibiotic resistance genes, with no reported cases of multidrug-resistant plasmids to date [36]. Although *E. bugandensis* R-18 demonstrated significant PGP traits under salt stress, its biosafety assessment in this study was limited to hemolytic activity screening. Since some *Enterobacter* species are opportunistic pathogens, further safety validations, including cytotoxicity assays, acute/chronic toxicity tests in model organisms, and genomic screening for virulence genes, are imperative prior to application.

Salt stress typically induces excessive Na^+^ uptake in plant roots, with Na^+^ being the primary ion responsible for ionic toxicity. Elevated cytoplasmic Na^+^ disrupts ion homeostasis, competitively inhibiting the absorption of essential mineral nutrients and leading to nutrient deficiency [37]. Additionally, salt stress impairs cellular metabolism, while excessive Na^+^/K^+^ ratios cause cellular dehydration, membrane dysfunction, and ion toxicity [38]. Studies have demonstrated a strong correlation between cytoplasmic K^+^ levels and plant salt tolerance: a higher K^+^/Na^+^ ratio corresponds to greater tolerance [39,40]. Plant-microbe symbiosis enhances plant survival under extreme conditions [41,42], and PGPB inoculation has proven effective in improving crop salt tolerance and mitigating soil salinization [43,44]. Many PGPB species maintained a high K^+^/Na^+^ ratio by altering the selective absorption of Na^+^, K^+^, and Ca^2+^ by plants, which can reduce the damage caused by salt stress to plants [45,46]. Therefore, maintaining a high level of K^+^/Na^+^ concentration was essential for normal cellular function and the growth and development of plants. This study revealed significant variations in K^+^ and Na^+^ levels across roots, stems, and leaves of maize seedlings under different treatments. Using the control group as a reference, the K^+^/Na^+^ ratio in the roots, stems, and leaves of maize seedlings under 100 mM NaCl stress was the lowest. The K^+^/Na^+^ ratio in the NaCl+R-18-treated group was significantly higher than that in the NaCl-treated group, with the most significant expression observed in the leaves. This indicated that under 100 mM NaCl conditions, a large amount of Na^+^ was absorbed, which stressed the growth of maize seedlings. Inoculating with strain R-18 can effectively prevent the absorption of Na^+^, playing a crucial role in balancing the K^+^/Na^+^ ratio in the roots, stems, and leaves of maize seedlings, thereby alleviating the damage caused by NaCl stress during the growth process of maize.

Chlorophyll plays a crucial role in photosynthesis in plants, and salt stress can have a negative impact on photosynthesis [47]. Cruz et al. [48] indicated that some salt-tolerant bacteria can increase chlorophyll content, and inoculating with ST-PGPB can alleviate the impact of salt stress on plant chlorophyll levels. Compared with the control group in this study, the chlorophyll content in the leaves of maize seedlings significantly decreased under NaCl stress. However, after inoculation with strain R-18, the chlorophyll content increased, which was beneficial for mitigating the negative impact of NaCl stress on photosynthetic pigments and enhancing photosynthesis. Another detrimental effect of salt stress is the increase in root cell osmotic potential, which restricts water uptake and disrupts plant metabolism [49,50]. Proline, as an important osmoregulatory substance in plants, plays a crucial protective role under salt stress [51]. This research found that proline content increased under NaCl stress, and it significantly increased after inoculation with strain R-18. Wang et al. [52] revealed that the content of soluble sugar and proline in maize seedlings significantly increased under salt stress, which could alleviate the effects of salt stress. Rashedy et al. [53] have shown that spraying proline solution on the leaves under salt stress could enhance the osmoregulatory function of pomegranate plants, thereby increasing yield. These results indicate that strain R-18 enhances osmoregulatory function by increasing proline content, thereby reducing NaCl-induced damage, improving salt tolerance, and significantly boosting maize yield (*p* < 0.05).

Several studies have demonstrated that the toxic effects of salinity may be mediated through the production of antioxidants and the inhibition of reactive oxygen species (ROS) generation [54]. Under salt stress, plants produced a large amount of ROS that caused oxidative damage to cells. These ROS attacked nucleic acids, proteins, and lipids within the plant, leading to the production of lipid peroxides such as MDA. MDA further induced lipid peroxidation of the cell membrane, resulting in increased membrane permeability and allowing a large amount of harmful substances to enter the cell. This ultimately disrupted the physiological and metabolic functions of the plant [55]. Research has shown that plants possess two primary mechanisms for degrading ROS: enzymatic and non-enzymatic pathways. The enzymatic mechanism involved antioxidants such as CAT, POD, and SOD [18]. To maintain the oxidative balance of cells, the antioxidant enzyme system in plants was activated, manifesting as increased SOD activity, which decomposed superoxide anion (O_2_^−^) into hydrogen peroxide (H_2_O_2_). The activities of POD and CAT also increase, further oxidizing and decomposing the produced H_2_O_2_ into water, thereby eliminating oxidative stress damage to cells [56]. In this research, due to NaCl stress, the MDA content in the leaves of maize seedlings was higher than that in the control group. Inoculating with strain R-18 reduced the MDA content. Under 100 mM NaCl stress, the SOD and POD activities in the leaves of maize seedlings in the NaCl-treated group were significantly higher compared to the control group. However, after inoculating with strain R-18, the SOD and POD activities in the leaves of maize seedlings were significantly lower compared to the NaCl-treated group. The increase in MDA led to the entry of harmful substances into the cells, disrupting the physiological and metabolic functions of plants. The enhancement of SOD and POD activities was aimed at eliminating oxidative stress damage to cells. Inoculating with strain R-18 reduced MDA content, SOD activity, and POD activity, indicating that strain R-18 significantly alleviated physiological damage to plants in vivo, thereby promoting maize growth (*p* < 0.05). However, under NaCl stress, the CAT activity in the leaves was significantly reduced. After inoculating with strain R-18, CAT activity increased. This restoration likely reflects microbial-mediated stimulation of the plant’s enzymatic defense system to scavenge excessive ROS under saline conditions, thereby enhancing plant stress tolerance and maintaining normal physiological metabolism [57]. Alternatively, this phenomenon may be attributed to the compartmentalized functionality of antioxidant enzymes, where CAT primarily neutralizes peroxisomal H_2_O_2_ while SOD and POD operate in other cellular organelles. From Figure 8, it can also be observed that under 100 mM NaCl conditions, the SOD activity, POD activity, and CAT activity of maize leaves after inoculating with strain R-18 showed a positive correlation trend with the control group, which was opposite to the trend in the NaCl-treated group (*p* < 0.05). These findings demonstrate that strain R-18 exhibited dual characteristics as both a plant growth-promoting bacterium and a microbial biostimulant.

## 5. Conclusions

This study identified *E. bugandensis* R-18 as a Gram-negative, salt-alkali tolerant plant growth-promoting bacterium possessing multiple plant-beneficial traits. Under 100 mM of NaCl stress conditions, the isolate R-18 inoculation effectively alleviated salt-induced growth inhibition in maize, improving all measured growth parameters while enhancing ion homeostasis through increased K^+^/Na^+^ ratios in roots, stems, and leaves. The isolate R-18 inoculation elicited coordinated physiological adaptations associated with improved salt tolerance in maize, characterized by enhanced chlorophyll and proline contents coupled with reduced MDA levels, demonstrating alleviated oxidative damage. The antioxidant system displayed a distinct regulatory pattern featuring decreased SOD and POD activities alongside increased CAT activity. *E. bugandensis* R-18 established an effective protective system that restricted excessive Na^+^ accumulation while maintaining optimal K^+^ assimilation in plant tissues. The synergistic integration of these growth-promoting and stress-mitigating responses demonstrates that *E. bugandensis* R-18 employs a dual-action mechanism to combat salinity stress, combining direct phytostimulation with systemic physiological regulation. However, the findings are exploratory, and rigorous biosafety assessments must precede any practical deployment. Future work should include genomic sequencing to identify potential pathogenic determinants, along with greenhouse and field trials under controlled biosafety protocols.

## Figures and Tables

**Table 1:** The bacteriological characteristics of strain R-18.

Test Indicators	Results	Test Indicators	Results
Gram stain	−	Cellulose hydrolysis	−
Methylic-red test	−	Nitrogen fixation	+
V–P test	+	Organophosphorus solubilizing	+
Catalase activity	+	Inorganic phosphorus	+
Citrate test	+	Potassium dissolving	+
Fluorescent pigment	+	Siderophore production	−
Hydrolysis of starch	+	ACC deaminase activity	+
Gelatin liquefaction	+	IAA production	+
Ammonia production test	+		

Note: “+” means that the strain has this function, and “−” means that it does not have this function.

## Data Availability

All original data generated in this study are included in the article, and the 16S rRNA gene sequence of the strain has been deposited in the National Center for Biotechnology Information (NCBI) database under accession number PQ814354.
